# A variance component based multi-marker association test using family and unrelated data

**DOI:** 10.1186/1471-2156-14-17

**Published:** 2013-03-04

**Authors:** Xuefeng Wang, Nathan J Morris, Xiaofeng Zhu, Robert C Elston

**Affiliations:** 1 Department of Biostatistics, Harvard School of Public Health, Boston, MA, 02115, USA; 2 Department of Epidemiology and Biostatistics, Case Western Reserve University, Cleveland, OH, 44106, USA

**Keywords:** Association studies, Family data, Score test, Multi-marker test

## Abstract

**Background:**

Incorporating family data in genetic association studies has become increasingly appreciated, especially for its potential value in testing rare variants. We introduce here a variance-component based association test that can test multiple common or rare variants jointly using both family and unrelated samples.

**Results:**

The proposed approach implemented in our R package aggregates or collapses the information across a region based on genetic similarity instead of genotype scores, which avoids the power loss when the effects are in different directions or have different association strengths. The method is also able to effectively leverage the LD information in a region and it can produce a test statistic with an adaptively estimated number of degrees of freedom. Our method can readily allow for the adjustment of non-genetic contributions to the familial similarity, as well as multiple covariates.

**Conclusions:**

We demonstrate through simulations that the proposed method achieves good performance in terms of Type I error control and statistical power. The method is implemented in the R package “fassoc”, which provides a useful tool for data analysis and exploration.

## Background

With the availability of cost-effective next generation sequencing platforms, one hot topic in the field is the analysis of low frequency and rare variants, which are believed to play an important role in the etiology of common complex diseases and may explain a portion of the missing heritability [1,2]. However, the sample sizes investigated in most studies are not large enough to ensure sufficient power for detecting rare variants with small or moderate effect sizes using single-marker tests [3]. Combining both family and unrelated data can improve statistical power over separate analysis of family data and unrelated data [4]. Current methods for testing rare variants are mainly based on aggregation or group tests that first pool together all variants with low minor allele frequencies in a region of interest and then test the association between phenotypes and the combined super-locus. Two of the earliest collapsing methods proposed are the combined multivariate and collapsing (CMC) test [5] and the weighted-sum method [6]. A number of variations of these methods have also been quickly developed [3,7-9]. Despite these developments, challenges remain to identify rare risk variants under different scenarios and assumptions. Because the aggregation test needs to assume homogeneity in the magnitude and direction of the individual effect sizes, it may experience massive loss in power when both protective and risk variants are present in the tested region, or when inappropriate weights/priors (or threshold of allele frequency) are used on rare variants. It is therefore timely to seek more powerful and reliable methods and designs. Motivated by recent works of Feng et al. [10] and Zhu et al. [11] who found that using sib pair data can increase power over using only unrelated samples, here we further explore the performance of methods with family information in searching for rare variants underlying complex traits.

In this work, we present an R package that implements a variance-component (VC) based association test that can test multiple common or rare variants jointly using both family and unrelated samples. The VC or linear mixed model (LMM) based approach aggregates or collapses the information across a region based on genetic similarity instead of genotype effects, which avoids the power loss when the effects are in different directions. A comparison study of binary traits [12] has also shown that the similarity-based test can be more powerful than the collapsing test when the rare variants have different association strengths. We propose to include an additional random effect in the mixed model in order to model polygenic effects and familial correlations.

Our method can readily allow adjusting for a non-genetic contribution to the familial similarity (shared environmental effects), as well as multiple covariates such as principal components of population structure. We compare the performance of the proposed method across a range of simulation scenarios with a fixed-effect or sum test based on Feasible Generalized Least Squares (FGLS). We also investigate the factors that influence power for testing rare variants. In this paper, we also show the connection between our method and kernel machine based methods [13,14], which may provide more flexibility in extending the proposed model. Although the simulations in this paper focus on rare variants analysis, our package can be readily applied to common variant association tests without any change.

## Implementation

Assume there are *q* subjects in the sample studied, including both family and unrelated individuals; and suppose for all individuals there is one gene or genetic region genotyped or sequenced that contains *n* variant sites or SNPs. For the *i*th individual, *y*_*i*_ denotes the observed quantitative trait value; *X*_*i*_ = (*x*_*i*,1_, *x*_*i*,2_ … *x*_*i*,*m*_) ' denotes an *m* × 1 vector of covariates (which might include sex, age, environmental factors, and principal components to allow for population stratification); *S*_*i*_ = (*s*_*i*,1_, *s*_*i*,2_, … *s*_*i*,*n*_) ' denotes an *n* × 1 genotype score vector for the *n* SNPs or variants in the region, coded 0, 1, or 2 (i.e., additive coding), reflecting the number of copies of the minor allele; and *Z*_*i*_ = (*z*_*i*,1_, *z*_*i*,2_ … *z*_*i*,*n*_) ' denotes a standardized genotype vector with the *ij*-th element $z_{i,j}=\left(s_{\mathrm{ij}}-2f_{j}\right)/\sqrt{2f_{i}\left(1-f_{j}\right)}$, where *f*_*j*_ is the minor allele frequency of the *j*th SNP or variant site.

### Linear mixed model and score test

The setup of our model is similar to the linear mixed model recently proposed to estimate the genetic variance explained by genome-wide SNPs [15,16], in which using all common SNPs was claimed to be able to uncover a large portion of missing heritability. That model required all subjects to be unrelated and assumed the similarity among individuals’ phenotype values is completely due to the similarity of their genetic components. The mixed model is written in matrix form as **y** = **Xβ** + **Wμ** + **ϵ**  with vary=WW'σu2+Iσe2, where y is a phenotype vector (assumed to be centered), X is a covariate matrix whose *i*th row is *X*_*i*_’, β is a vector of coefficients (fixed effects) for covariates X, μ is a vector of causal variant effects with $\mu \sim N\left(0,I\sigma_{\mu }^{2}\right)$, W is a standardized genotype matrix, I is an identity matrix and ϵ is a random error vector with $\epsilon \sim N\left(0,I\sigma_{\epsilon }^{2}\right)$. In the real case when the position and number of causal variants are unknown, a working model can be represented as **y** = **Xβ** + **δ** + **ϵ**, where δ is a vector representing random effects of all SNPs, with $\delta \sim N\left(0,Α\sigma_{\delta }^{2}\right)$ and thus $\mathrm{var}\left(y\right)=A\sigma_{\delta }^{2}+I\sigma_{\epsilon }^{2}$. A can be interpreted as the genetic relationship matrix (GRM) among individuals and its *jk*-th element is $A_{\mathrm{jk}}=\sum_{i=1}^{N}\frac{\left(s_{\mathrm{ij}}-2f_{i}\right)\left(s_{\mathrm{ik}}-2f_{i}\right)}{2f_{i}\left(1-f_{i}\right)}/N$, where N is the total number of genome-wide SNPs. The variance components can be estimated via the restricted maximum likelihood (REML) method [16].

To estimate and test the variance expressed by a gene or a genomic region using both family and unrelated data, intuitively one can extend the above model by

(1) y=Xβ+Zγ+δ+ε=Xβ+g+δ+ε,

where γ is a vector of the random effect of SNPs in the studied region distrtibuted $\sim N\left(0,I\sigma_{\gamma }^{2}\right)$, Z is the standardized genotype matrix, and δ is a vector of random effect representing the polygenic genic effects over the genome. Under this model, the marginal phenotypic variance V_y_ can be partitioned into components attributable to the SNPs in the studied region, polygenic and residual variances:

(2) Vy=Vg+Vδ+Vε=ZZ'σγ2+Aσδ2+Iσε2=Sσg2+Aσδ2+Iσε2,

where **S** = **ZZ** '/*n*, and $\sigma_{g}^{2}$ represents the variance explained by the SNPs in the region, i.e., $\sigma_{g}^{2}=n\sigma_{\gamma }^{2}$. A and S can thus be interpreted as two genetic similarity matrices. In this model, if two individuals are from different families (unrelated), their corresponding entry in A is calculated genomewide in the same way as above, but excluding the SNPs in the region we are testing. For individuals in the same family, or when the genome-wide SNPs are not available, the corresponding entries in A can be approximately computed by twice their kinship coefficients - which depends only on the relatedness between individuals - in which case $V_{y}=S\sigma_{g}^{2}+2\Phi \sigma_{\delta }^{2}+I\sigma_{\epsilon }^{2}$ , where Φ denotes the q × q kinship matrix. To account for the common environmental factors shared by family members, we can include a common environmental factor in the model. Our mixed linear model now becomes **y** = **Xβ** + **g** + **δ** + **α** + **ϵ**  with $V_{y}=S\sigma_{g}^{2}+A\sigma_{\delta }^{2}+C\sigma_{\alpha }^{2}+I\;\sigma_{\epsilon }^{2}$, where a is the effect due to the shared common environment factors with $\alpha \sim N\left(0,C\sigma_{\alpha }^{2}\right)$ and C is a matrix with the the *jk*-th element being 1 if the *j*-th and *k*-th individuals belong to the same family and 0 otherwise. Note that, by adding a variance component common to siblings, it is also easy to allow for the fact that siblings resemble each other more than do parents and their offspring, whether due to dominant effects or common environment.

This model can be readily applied to haplotype-based analysis with the design matrix for genotype scores Z replaced by a haplotype matrix H, where a vector H_*i*_ records the *i*-th individual’s haplotype pair via a given scoring rule [17]. Hence, Model (1) becomes **y** = **Xβ** + **Hγ**_*h*_ + **δ** + **ϵ**  with $V_{y}=S_{h}\sigma_{h}^{2}+A\sigma_{\delta }^{2}+I\;\sigma_{\epsilon }^{2}$, where γ_*h*_ represents the random effect of haplotypes; S_*h*_ is a matrix of pair-wise similarity scores between the haplotype pairs of two individuals, with the *ij*-th element equal to $\sum_{h,k}H_{i,h}H_{j,k}\times s\left(h,k\right)$[18], where *s*(*h*, *k*) is a similarity matrix measuring the similarity between haplotypes *h* and *k*. If we set s(*h*, *k*) as the proportion of matching alleles between two haplotypes, the *ij*-th element of S_*h*_ will be equivalent to the average allelic sharing across multiple markers between two individuals and thus phase information is not required.

Our primary interest lies in detecting whether there is an effect of a genomic region on the phenotype, which is assessed by testing the null hypothesis $H_{0}:\sigma_{g}^{2}=0.$ In the following, we construct a fast score test based on the MLE and REML framework as an extension of that proposed by Tzeng and Zhang [17]. For the sake of demonstration, we first assume there is no shared environmental effect within families. Assuming a normally distributed quantitative trait, the log-likelihood function and its REML version for the variance component model are written as

$$\begin{array}{c} \ell \;\left(\sigma_{g}^{2},\sigma_{\delta }^{2},\sigma_{e}^{2};y\right)=-\frac{1}{2}\log\left|V\right|-\frac{1}{2}\log\left|X^{T}V^{-1}X\right|\\ \;-\frac{1}{2}{\left(y-\mathrm{X\beta}\right)}^{T}V^{-1}\left(y-\mathrm{X\beta}\right) \end{array}$$

$$\begin{array}{c} \ell_{\mathrm{REML}}\left(\sigma_{g}^{2},\sigma_{\delta }^{2},\sigma_{e}^{2};y\right)=-\frac{1}{2}\log\left|V\right|-\frac{1}{2}\log\left|X^{T}V^{-1}X\right|\\ \;-\frac{1}{2}y^{T}P^{-1}y, \end{array}$$

where $V=S\sigma_{g}^{2}+A\sigma_{\delta }^{2}+I\;\sigma_{\epsilon }^{2}$, and **P** = **V**^− 1^ − **V**^− 1^**X**(**X**^*T*^**V**^− 1^**X**)^− 1^**X**^*T*^**V**^− 1^ is the projection matrix under the linear mixed model (1).

It will be convenient to denote the parameter of interest $\sigma_{g}^{2}$ by *τ*, and the nuisance parameters $\left(\beta ,\sigma_{\delta }^{2},\sigma_{e}^{2}\right)$ by *η*. Under the null hypothesis, the score statistic with respect to *τ* is given by

$$\begin{array}{l} U_{\tau }\left(\hat{\eta }\right)=\frac{\partial \ell \left(\tau ,\eta ;y\right)}{\partial \tau }|{}_{\tau =0,\eta =\hat{\eta }}\\ \;=-\frac{1}{2}\mathrm{tr}\left(V_{0}^{-1}S\right)+\frac{1}{2}{\left(y-X\hat{\beta }\right)}^{T}V_{0}^{-1}SV_{0}^{-1}\left(y-X\hat{\beta }\right) \end{array}$$

where $V_{0}=A{\hat{\sigma }}_{\delta }^{2}+I\;{\hat{\sigma }}_{\varepsilon }^{2}$, and $\hat{\eta }=\left(\hat{\beta },{\hat{\sigma }}_{p}^{2},{\hat{\sigma }}_{e}^{2}\right)$ is the maximum likelihood estimate of *η* under the null linear mixed model **y** = **Xβ** + **δ** + **ε**. These estimates can be obtained using the regular statistical software that implement mixed-model functionality, or even more easily in some genetic analysis packages that can directly read in a kinship matrix, such as EMMA [19] (http://mouse.cs.ucla.edu/emma/) and GenABEL (http://www.genabel.org/).

However, the asymptotic distribution of the above score statistic is not a typical standard normal distribution (neither does the corresponding LRT statistic converge to a mixture of $\chi_{0}^{2}$ and $\chi_{1}^{2}$). This is because, in contrast to IBD, the genotype-based similarity matrix **S** = **ZZ** '/*n* does not have a block diagonal structure, and thus the statistic cannot be written in a form of a sum of independent variables that meets the asymptotical conditions indicated in Lin [20]. Instead, we can construct the test on the basis of the second term of $U_{\tau }\left(\hat{\eta }\right)$, following the approach proposed by Zhang and Lin [21]. Letting $M=\frac{1}{2}V_{0}^{-1}SV_{0}^{-1}$ and $\tilde{y}=V_{0}^{-1/2}\left(y-X\hat{\beta }\right)$, the new statistic becomes

$$T_{\tau }={\left(y-X\hat{\beta }\right)}^{T}M\left(y-X\hat{\beta }\right)={\tilde{y}}^{T}V_{0}{}^{1/2}MV_{0}{}^{1/2}\tilde{y},$$

Because asymptotically $\tilde{y}\sim N\left(0,I\right)$, *T*_*τ*_ follows a weighted sum of chi-square variables: $T_{\tau }∼\sum_{i=1}^{m}\lambda_{i}\chi_{1i}^{2}$, where $\chi_{1i}^{2}$ are independent chi-square random variables with one degree of freedom, and the weights *λ*_*i*_ are the *i*-th ordered nonzero eigenvalues of **V**_0_^1/2^**MV**_0_^1/2^. A good approximation may be obtained using only *r* (<<*q*) dominant eigenvalues as *λ* usually decays rapidly toward zero.

Significance of a test can be evaluated empirically through simulating a large set of sums of chi-squared random variables, where the *p*-value is obtained by calculating the proportion of the generated random variables that are greater than the observed statistic. However, this is considerably slower than computing the eigenvalues of **V**_0_^1/2^**MV**_0_^1/2^ when the sample size is large. Furthermore, to ensure reliable results for a large effect size or small α level, one needs to run a huge number of simulations. For instance, when α is set at 1 × 10^-5^, at least 10^7^ simulations are needed for each test. This becomes computationally infeasible for a genome-wide scan. Here we consider Satterthwaite’s procedure to approximate the null distribution of *T*_*τ*_ by a scaled chi-square distribution $k\chi_{\upsilon }^{2}$ or a gamma distribution Gamma (*a*, *b*). The two parameters in the approximate distribution are calculated by matching the first and second moments (mean and variance) with those of the score statistic. Taking a Gamma distribution as an example, we attempt to obtain $\mathrm{ab}=\mu_{T}\;\mathrm{and}\;ab^{2}=\nu_{T}\Leftrightarrow \;a=\mu_{T}^{2}/\nu_{T}\;\mathrm{and}\;b=\nu_{T}/\mu_{T}\;$. Due to its quadratic form, it is easy to obtain the mean and variance of *Tτ*:

$$\begin{array}{c} \mu_{T}=E\left({\tilde{y}}^{T}V_{0}{}^{1/2}MV_{0}{}^{1/2}\tilde{y}\right)\\ \;=\mathrm{tr}\left(V_{0}{}^{1/2}MV_{0}{}^{1/2}\right)=\frac{1}{2}\mathrm{tr}\left(V_{0}{}^{-1}S\right)\\ \;\nu_{T}=\mathrm{var}\left({\tilde{y}}^{T}V_{0}{}^{1/2}MV_{0}{}^{1/2}\tilde{y}\right)\\ \;=2\mathrm{tr}\left[{\left(V_{0}{}^{1/2}MV_{0}{}^{1/2}\right)}^{2}\right]=\frac{1}{2}\mathrm{tr}\left[{\left(V_{0}{}^{-1}S\right)}^{2}\right] \end{array}$$

To account for the fact that the nuisance parameters *η* are estimated and replaced by their MLEs $\hat{\eta }$, *v*_*T*_ may be replaced by the partial information $I_{\tau }=I_{\tau \tau }-I_{\tau \eta }I_{\eta \eta }^{-1}I_{\eta \tau }$ (to subtract the loss of information in the data due to *η* being unknown), where $I_{\tau \tau }=\frac{1}{2}\mathrm{tr}\left[{\left(V_{0}{}^{-1}S\right)}^{2}\right]$, $I_{\tau \eta }=\frac{1}{2}\mathrm{tr}\left[V_{0}{}^{-1}SV_{0}{}^{-1}\frac{\partial V}{\partial \eta }\right]$, $I_{\eta \eta }=\frac{1}{2}\mathrm{tr}\left[V_{0}{}^{-1}\frac{\partial V}{\partial \eta }V_{0}{}^{-1}\frac{\partial V}{\partial \eta }\right]$ and $I_{\eta \tau }=I_{\tau \eta }^{T}$. When the estimation and score test is based on the REML, the above formulas remain the same but with **V**_0_^− 1^ replaced by the projection matrix **P**_0_ = **V**_0_^− 1^ − **V**_0_^− 1^**X**(**X**^*T*^**V**_0_^− 1^**X**)^− 1^**X**^*T*^**V**_0_^− 1^.

Satterthwaite’s procedure is fairly fast but may not have desirable performance in the extreme tails of the distribution. An alternative procedure would be to fit a distribution for which the first three moments are estimated, rather than only the first two. Possibilities would be to assume the distribution is a multiple of a non-central chi-square distribution, estimating the multiple and the two parameters of the non-central chi-square distribution from the empirical first three moments; alternatively, one could fit a distribution that is a multiple of a power of a chi-square distribution, estimating the multiple, the power and the *d.f.* from the first three moments. A similar strategy of utilizing higher moments/cumulants has been proposed by Liu et al. [22], in which the parameters of the approximate distribution are determined in such a way that skewness is matched while the difference in kurtosis is minimized. Other possible methods include the Davies method [23] (based on numerical inversion of the characteristic function), Farebrother’s [24] and Imhof’s methods [25,26]. These methods are available in an R package called “CompQuadForm”.

The VC score approach described above has a special advantage of being easily extended to, and compatible with, the kernel machine regression that allows for more flexible modeling of genetic effects. Methods like least-square kernel machines (LSKM) and their variants have been successfully applied in multi-marker association tests with both common and rare variants [13,14,27]. Under the framework of LSKM, the outcome of an individual can be described by the following semiparametric regression model:

$$y_{i}=x_{i}^{T}\beta +h\left(s_{i}\right)+\delta_{i}+\varepsilon_{i}\;,$$

where *h*(.) is a nonparametric smoothing function that allows a flexible modeling of the influence of the genotype information s_*i*_ on the trait value. The function space that *h*(.) lies in is fully determined by a positive semidefinite kernel function *K*(.,.). A kernel function can implicitly map input data to a higher-dimension inner product space, and thus defines the complexity level of the relationship between the genotypes and the trait. Intuitively, a kernel function *K*(s_*i*_,s_*j*_) can also be thought as a similarity measure between the genotypes of individuals *i* and *j* (in the genomic region tested). Three types of kernel used most often are the linear, quadratic and Gaussian kernels. Note that the linear kernel $K\left(s_{i},s_{j}\right)=s_{i}^{T}s_{j}$ will be analogous to a covariance when s is centered. One can choose an appropriate non-linear kernel to accommodate interaction and nonlinear genetic effects.

Given the close relationship between the LSKM and GLMM framework, Liu et al. [28] found that it is much more convenient to test the null hypothesis *H*_0_ : *h*(**z**) = 0 based on the related linear mixed model. The corresponding model in our method is

y=Xβ+h+δ+ε,

where h is regarded as a random effect with mean zero and variance τ**K**, where **K** is an *n* × *n* matrix with the *ij*-th element equal to *K*(s_i_,s_*j*_). It can be shown that the best-linear unbiased estimators (BLUP) of h and β have the same form as those derived via LSKM estimation [27]. The equivalence implies that we can directly use the above likelihood functions and the test procedures that are constructed on Model (1), but with g replaced by h, and the similarity matrix **S** replaced by **K**.

To accommodate rare variant SNPs, a weighted kernel function might be used so that similarity in rare variants will be emphasized. Assuming additive genotypic coding, a weighted IBS kernel can be written as $K\left(s_{i},s_{j}\right)=\sum_{l=1}^{p}w_{l}\left(2-\left|s_{i,l}-s_{j,l}\right|\right)$. One such weight is $w_{l}=1/\sqrt{p_{l}}$, where *p*_*l*_ is the minor allele frequency of the *l*^th^ SNP or variant. A more flexible way is based on the density function of a beta distribution: *w*_*l*_ = *Beta*(*p*_*l*_; *a*, *b*) [14]. Note that, when *a = b =* 0.5, *w*_*l*_ will be equal to 1/*p*_*l*_(1 − *p*_*l*_), in which case the weighted IBS kernel with the original genotype scores will be analogous (but not exactly identical) to using standardized genotypes in model (1). Under this formulation, the VC score test can be viewed as a special case of the LSKM approach.

### Simulations

We performed simulation studies to examine the type I error and power of the proposed score approach for detecting genetic variants under a range of scenarios, especially when rare variants are the cause of the phenotypic variation. We began by simulating 10,000 haplotypes of a 500 kb genomic region under the coalescent model using the software “cosi” (http://www.broadinstitute.org/~sfs/cosi/), with an effective population size of 10^4^, mutation rate set at 1.5 × 10^-8^ per bp per generation, and the recombination rate varying across the region with a local window size of 100 kb. A total of 2883 variant locations were generated using this setting, of which 73 % had minor allele frequencies < 0.05. We randomly picked a region of 500 variants as our test region. In determining causal variants and risk haplotypes, we used a procedure similar to that described in Feng et al. [10]. Specifically, we assumed that only the variants with MAF < 2% can be causal variants, and considered a collapsing risk model in which the risk of one haplotype is determined by the presence of a minor allele at any risk location within the region. We then randomly drew causal variants from the pool of locations with MAF <2% until the accumulated frequency of risk haplotypes reached 10%. In each simulation, this procedure led to around 5%-8% of the variants being risk variants. In other words, we considered as risk haplotypes those that include at least one causal variant and assumed that their contributions to the phenotype are identical, i.e., the phenotype of an individual depends upon a genomic region only through the number of risk haplotypes she/he carries. The genotypes of unrelated individuals or those of founders in family data were simulated by randomly sampling with replacement two haplotypes from the 10,000 haplotypes. The haplotype data within one individual were then combined and converted into unphased genotype data. For illustration purposes, we only considered nuclear families for family data in our simulations, in which the number of children in each family was a random number drawn from a Poisson distribution with mean λ = 2. To simulate the genotypes of the second generation, we randomly drew one of the two haplotypes from each parent and then transmitted them to his/her offspring.

We determined the quantitative trait values based on a normal distribution. Specifically, we first calculated the causal genetic score (g) of an individual by g = *zu*, where *u* is the effect size and z_*i*_ is coded as 0, 1, or 2 indicating the number of risk haplotypes. Next we generated the overall residual variance by var(*g*)(1/*h*^2^ − 1), in which *h*^2^ is the proportion of phenotypic variance explained by a genomic region, and var(g) is the theoretical variance of the genetic score calculated as var(*g*) = var(*z*)*u*^2^ − 2*r*(1 − *r*)*u*^2^, where *r* is the proportion of risk haplotypes (in 10,000 samples). The variances of the polygenic effect (*p*) and random error effect (*e*) were split from the overall residual variance to meet two conditions: var(*g*) + (*p*) = 0.4 and var(*e*) = 1-var(*g*)-var(*p*). For founders and unrelated individuals, we generated values of *g*, *p*, and *e* from normal distributions with means zero and variances var(*g*), var(*p*) and var(*e*), respectively. For children, *p* was generated by $p_{c}=\frac{1}{2}\left(p_{m}+p_{f}\right)+\frac{1}{\sqrt{2}}p_{i}$, where *p*_*m*_ and *p*_*f*_ are the values of the parents and *p*_*i*_~*N*(0, var (*p*)). The phenotypic value of each individual was then calculated as *y* = *g* + *p* + *e*, and all *y* were centered before any analysis. For simplicity, here we did not simulate covariates or shared environment effects.

We designed various simulation scenarios by changing parameters such as *h*^2^, sample sizes, and the proportion of risk haplotypes. Each set-up consisted of 200 independent replications (by updating each time not just phenotypes, but also genotypes). To compare with fixed-effect sum tests, each data set was also analyzed by the feasible generalized least squares regression model (FGLS). FGLS is very similar to generalized least squares except that it uses an estimated variance-covariance matrix (which can be obtained under the null model) [29]. We used the ‘FGLS’ function in the R package “MixABEL” (http://www.genabel.org/packages/MixABEL) to implement this analysis.

**Figure 1:**
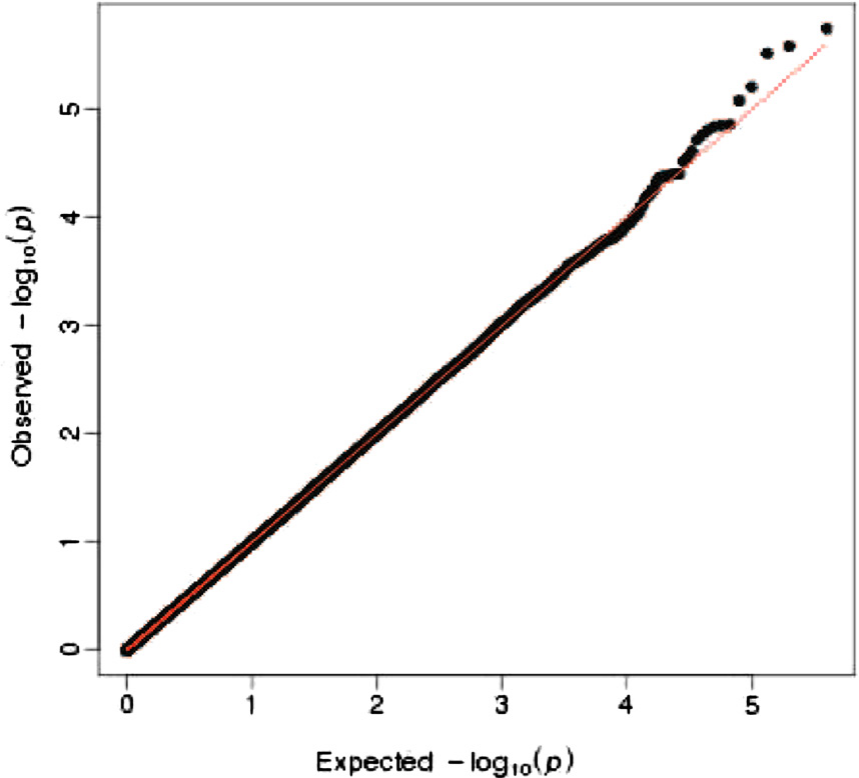
Quantile-quantile plot comparing empirical p-values (based on 400,000 simulations under the no assocation model) against those expected under the null.

We have evaluated the type I error for the proposed method by generating 400,000 replicates under the H_0_. Figure 1 shows a quantile-quantile plot of observed p values against those expected under the null.

## Results and discussion

In our primary set of simulation for power comparison, 500 nuclear families and 2,000 unrelated individuals were generated, based on the simulation procedures described above, where the proportion of phenotypic variance explained by a region was set at (0, 0.01,…, 0.05). Each data set was analyzed by four different strategies: (1) the proposed VC-score test with all 500 variants; (2) the FGLS test using the genotype sum of 500 variants; (3) the VC-score test with only rare variants (with minor allele frequency (MAF) < 0.02 in the sample) included; (4) the FGLS test using the genotype sum of rare variants. Because we used standardized genotypic scores and true MAF thresholds for rare variants, results from method (4) should represent the best results that a weighted-sum aggregation test could possibly reach. The power was assessed at the 0.05 and 1 × 10^-6^ significance levels using 200 replications. When α was set at 0.05 and *h*^2^ (heritability) set at 0, all analysis strategies maintain type I error rates around the nominal level. The power of the VC-score test is close to or higher than the FGLS method under all scenarios. The VC-score method also demonstrated great robustness to the number of noise markers. Results indicate that excluding common variants (all non-causal) results in noticeable power increase when using the FLGS method, but has nearly no effect on the VC method. We also tried the VC method using the genotype sum of rare variants only. Results are not presented here because they are exactly the same as those from method (4) in view of the equivalence of the two statistics when the genotype sum is used.

The simulation results indicate that, under the current simulation settings and sample sizes, the proposed method will have adequate power to detect a genomic region with *h*^2^ around 0.01 in a candidate gene analysis, or a region with *h*^2^ around 0.02 in a genome-wide scan. Table 1 summarizes the results from the simulations with increasing sample sizes, in which the power was evaluated at significance levels of .05, 1 × 10^-5^, and 1 × 10^-6^, respectively. Three different designs were considered. In design I we included an additional 1,000 unrelated individuals, while in design II we added another 250 families (approximately the same genotyping effort as 1000 unrelated individuals). Both designs gave apparent power increase compared to previous simulations (around 15% more when *h*^2^ is below 0.03), but the increase in design I is slightly greater than that in design II. Our preliminary simulations show that the difference can be more significant when using a smaller base sample size. As generally accepted, an association analysis using related individuals is less informative than one using the same number of unrelated individuals, and is thus less powerful. In practice, families are not randomly sampled but often selected through probands or because of existing linkage evidence. We explored this effect in design III. Rather than going through the complex modeling of the ascertainment process, we created an enriched risk haplotype pool by directly removing 2,000 non-risk haplotypes. Therefore, each risk haplotype has a little more than 1/8 chance to be assigned to a family founder instead of about 1/10. As shown in Table 1, design III had much better performance than design I.

**Table 1:** Power of VC-score tests under different sample sizes

**Design**	**h**^**2**^	**Significance level (α)**		
		**0.05**	**1 × 10**^**-5**^	**1 × 10**^**-6**^
I. 500/3000				
	0.01	0.840	0.355	0.270
	0.02	1	0.855	0.780
	0.03	1	0.985	0.980
II. 750/2000				
	0.01	0.890	0.345	0.235
	0.02	1	0.825	0.725
	0.03	1	0.975	0.970
III. 750^a^/2000				
	0.01	0.94	0.435	0.335
	0.02	1	0.92	0.880
	0.03	1	1	0.990

Note.—The design column indicates # of families / # of unrelated individuals. Only nuclear families are simulated, with each family having two parents with a mean of two children.

a. 750 families simulated with enriched risk haplotypes.

We also indirectly compared the performance of the VC and FGLS methods by varying parameters that can affect the effect sizes. We calculated the power of the two methods when the proportion of risk haplotypes was set at 5%, i.e., only 500 haplotypes were tagged as risk in the 10,000 haplotype pool. Although each individual has less chance to carry a risk haplotype, there would be fewer causal variants with larger effect size simulated (if the variance explained by a region is fixed). It was found that both methods had substantial power increase compared to the first simulation, but the VC method had greater improvement than the FGLS. In a simulation set-up where causal SNPs (rare variants only) were not assigned independently (but pairs of SNPs close to each other, and thus correlated, were selected), we found the VC method had a slight power improvement while the FGLS had a small loss in power. Detailed results are listed in Additional file 1. In this work, we did not simulate data with a polygenic term but analyzed the data ignoring it because the results from such a comparison are quite predictable. Because the polygenic terms are correlated among individuals within a family, ignoring such correlations in the analysis will cause a deflated type I error rate and thus render any power comparison invalid.

Many extensions are possible for improved implementation of the proposed model and testing procedure. This method can be easily extended to incorporate nonlinear and interaction effects. As discussed previously, our method can be considered as a special case in the framework of the kernel machine method. Interaction and nonlinear effects among markers can be further included in the model through specifying a valid kernel function or similarity metric. Also, more flexible weights may be incorporated into the kernel function according to allele frequencies or other prior information. Although a normally distributed trait was assumed throughout this study, the derived score statistic is also appropriate for non-normal traits [17]. For binary traits, we can construct the score test analogously, based on the logistic version of the mixed variance model (1) with the outcome *y* replaced by logit[P(*y* = 1)], or via extending the logistic kernel machine method [13]. When there are several correlated traits available, the multivariate variance component model will be very useful because it can have more power than univariate analysis.

## Conclusions

We propose a multi-marker VC-based association test using both family and unrelated data. A fast score test has been built on the ML and REML framework, in which only the parameters in the null model need to be estimated. Owing to the non-block-diagonal structure of the genotype-based similarity matrix, the score statistic derived has a different form from that based on the typical VC model for linkage analysis. We demonstrate through simulations that the proposed method achieves good performance in terms of Type I error control and statistical power. The method is implemented in the R package “fassoc”. We believe that “fassoc” will be a useful tool to complement existing software for family-based association studies.

## Availability and requirements

Project name: fassoc package

Project home page: https://r-forge.r-project.org/R/?group_id=1379

Operating system(s): Linux, Mac OS X, Windows

Programming language: R

Other requirements: R (≥2.15.1)

License: GNU GPL

Any restrictions to use by non-academics: none except those posed by the license

## Competing interests

The authors declare that they have no competing interests.

## Authors’ contributions

XW and NJM participated in the design of the study and implementation of the method. XW drafted the manuscript. XZ and RCE participated in the conception and design of the study and in editing the manuscript. All authors read and approved the final manuscript.

## Supplementary Material

Additional file 1 Additional simulation results and software. Click here for file
