# Supplementary material for: A variance component based multi-marker association test using family and unrelated data
Source: BMC Genet. 2013 Mar 4;14:17. doi: 10.1186/1471-2156-14-17 (PMC3614458; doi:10.1186/1471-2156-14-17)
Supplement: Additional file 1 — Additional simulation results and software. [file 1471-2156-14-17-S1.docx]

**Supplementary Information**

**A variance component based multi-marker association test using family and unrelated data**

Xuefeng Wang, Nathan J. Morris, Xiaofeng Zhu, and Robert C. Elston

# CONTENTS

Software description Page 2

Supplementary tables 1-3

1. Power comparisons I Page 3
2. Power comparisons II Page 4
3. Power comparisons III Page 5

Computation time, reliability and efficiency Page 6

# Software description

**Download page**: <https://r-forge.r-project.org/R/?group_id=1379>

**Operating system(s):** Linux, Mac OS X, Windows

**Programming language**: R

**Other requirements:** R (≥2.15.1)

**License:** GNU GPL

**Restrictions to use by non-academics:** none except those posed by the license

**Install**:

Start R, then type install.packages("fassoc", repos="http://R-Forge.R-project.org")

**Key functions**

*vc.score*: performs marker set based association (score test) based on the proposed variance component model.

*fgls:* performs marker set based association based on the Feasible Generalized Least Squares (FGLS) model.

*ped.simu*: This function simulates both phenotypes and genotypes of a combination of family and unrelated data.

Details on usage of these functions can be found in the R package manual.

**Limitations:**

The current implementation is not able to incorporate shared environmental effects and nonlinear (interaction) effects. The main testing function in this package uses a standardized genotype as input (i.e., fixed weights on each genetic marker) and does not allow for user-specified weights.

# Supplementary Table 1.

Power comparisons between the VC-score and FGLS methods using the four analysis strategies :1) the VC-score test, with all SNPs included; 2) the FGLS test, with all SNPs included; 3) the VC-score with only rare variants included; 4) the FGLS testing using only rare variants included. Power is estimated using 500 replicates at levels of 0.05, 1×10^-5^, 1×10^-6^, respectively. The simulations are based on 500 nuclear families and 2,000 unrelated individuals.

| Methods | h^2^ | Significance level (α) | | |
| --- | --- | --- | --- | --- |
|  |  | 0.05 | 1×10^-5^ | 1×10^-6^ |
| 1. VC-score (all markers) |  |  |  |  |
|  | 0.01 | 0.758 | 0.052 | 0.012 |
|  | 0.02 | 0.98 | 0.348 | 0.186 |
|  | 0.03 | 1 | 0.764 | 0.592 |
| 1. FGLS (all markers) |  |  |  |  |
|  | 0.01 | 0.702 | 0.038 | 0.016 |
|  | 0.02 | 0.938 | 0.252 | 0.128 |
|  | 0.03 | 0.982 | 0.488 | 0.358 |
| 1. VC-score (rare only) |  |  |  |  |
|  | 0.01 | 0.838 | 0.026 | 0.01 |
|  | 0.02 | 0.994 | 0.294 | 0.144 |
|  | 0.03 | 1 | 0.688 | 0.478 |
| 1. FGLS (rare only) |  |  |  |  |
|  | 0.01 | 0.852 | 0.096 | 0.034 |
|  | 0.02 | 0.97 | 0.464 | 0.292 |
|  | 0.03 | 0.994 | 0.782 | 0.646 |

# Supplementary Table 2.

Comparisons of the power for two additional scenarios described in the paper. (a) The proportion of risk haplotypes was set at 5%. (b) Causal variants are correlated. Power is estimated using 500 replicates at levels of 0.05, 1×10^-5^, 1×10^-6^, respectively. The simulations are based on 500 nuclear families and 2,000 unrelated individuals.

| Methods | h^2^ | Significance level (α) | | |
| --- | --- | --- | --- | --- |
|  |  | 0.05 | 1×10^-5^ | 1×10^-6^ |
| 1. VC-score (hrisk=0.05) |  |  |  |  |
|  | 0.01 | 0.826 | 0.18 | 0.094 |
|  | 0.02 | 0.972 | 0.678 | 0.544 |
|  | 0.03 | 0.998 | 0.858 | 0.816 |
| 1. FGLS (hrisk=0.05) |  |  |  |  |
|  | 0.01 | 0.700 | 0.102 | 0.042 |
|  | 0.02 | 0.862 | 0.384 | 0.266 |
|  | 0.03 | 0.872 | 0.67 | 0.524 |
| 1. VC-score (correlated causal) |  |  |  |  |
|  | 0.01 | 0.762 | 0.036 | 0.016 |
|  | 0.02 | 0.988 | 0.318 | 0.182 |
|  | 0.03 | 0.998 | 0.756 | 0.616 |
| 1. FGLS (correlated causal) |  |  |  |  |
|  | 0.01 | 0.716 | 0.031 | 0.011 |
|  | 0.02 | 0.908 | 0.216 | 0.114 |
|  | 0.03 | 0.974 | 0.526 | 0.340 |

# Supplementary Table 3.

Comparisons of the power based on a new simulation scheme where ten causal markers are simulated. The phenotypic value of each individual was calculated from $y_{i}=\sum_{k=1}^{10} s_{k}b_{k}+g_{i}+e_{i}$, where $s_{k}$ is the genotypic value of the *k*th causal markers, $b_{k}$is the effect size of the causal marker. $g_{i}$ and $e_{i}$ are simulated the same as described in the paper. In the first simulation setting, all $b_{k}$are set to be 0.5. In the second simulation, half of the effect sizes are set to be 0.5, and half to be -0.5. These two simulations are based on 500 nuclear families and 1,000 unrelated individuals.

| Methods | h^2^ | Significance level (α) | | |
| --- | --- | --- | --- | --- |
|  |  | 0.05 | 1×10^-5^ | 1×10^-6^ |
| 1. VC-score (Simulation I) |  |  |  |  |
|  | 0.01 | 0.888 | 0.166 | 0.092 |
|  | 0.02 | 0.996 | 0.582 | 0.448 |
|  | 0.03 | 0.998 | 0.87 | 0.806 |
| 1. FGLS (Simulation I) |  |  |  |  |
|  | 0.01 | 0.394 | 0.034 | 0.010 |
|  | 0.02 | 0.450 | 0.098 | 0.066 |
|  | 0.03 | 0.568 | 0.186 | 0.138 |
| 1. VC-score (Simulation II) |  |  |  |  |
|  | 0.01 | 0.862 | 0.116 | 0.052 |
|  | 0.02 | 0.984 | 0.572 | 0.446 |
|  | 0.03 | 0.998 | 0.832 | 0.75 |
| 1. FGLS (Simulation II) |  |  |  |  |
|  | 0.01 | 0.234 | 0.008 | 0 |
|  | 0.02 | 0.338 | 0.056 | 0.028 |
|  | 0.03 | 0.412 | 0.082 | 0.058 |

# Computation time, reliability and efficiency

Speed: The computation time of the score test function implemented in the software depends on the number of markers and sample sizes, especially the number of related individuals. Our tests were run on a 2.67 GHz Xeon, and with 500 markers in a gene region.

Reliability and efficiency: The current software provides a limited level of error-control. The input file format must strictly follow what we stated in the help file and in the example datasets. This package relies on an R function in the EMMA package to estimate the variance component parameters under the null. The computational efficiency can be largely improved in a genome-wide scan analysis, where the null parameters only need to be estimated once.

| Size of related individuals | Size of unrelated individuals | Time (s) |
| --- | --- | --- |
| 400 | 500 | 2.4 |
| 400 | 1000 | 10.4 |
| 400 | 2000 | 44.7 |
| 1000 | 500 | 10.8 |
| 1000 | 1000 | 30.2 |
| 1000 | 2000 | 85.8 |
| 2000 | 500 | 55.9 |
| 2000 | 1000 | 94.6 |
| 2000 | 2000 | 200.7 |
